# A Holey Graphene Additive for Boosting Performance of Electric Double-Layer Supercapacitors

**DOI:** 10.3390/polym12040765

**Published:** 2020-04-01

**Authors:** Jun-Bin Huang, Jagabandhu Patra, Ming-Hsien Lin, Ming-Der Ger, Yih-Ming Liu, Nen-Wen Pu, Chien-Te Hsieh, Meng-Jey Youh, Quan-Feng Dong, Jeng-Kuei Chang

**Affiliations:** 1 Department of Chemical and Materials Engineering, Chung Cheng Institute of Technology, National Defense University, 1000 Xingfeng Road, Taoyuan 335, Taiwan; a0956923012@gmail.com (J.-B.H.); mslin479@gmail.com (M.-H.L.); liuym@ndu.edu.tw (Y.-M.L.); 2 Hierarchical Green-Energy Materials (Hi-GEM) Research Center, National Cheng Kung University, 1 University Road, Tainan 70101, Taiwan; jpatra2014@gmail.com; 3 Department of Materials Science and Engineering, National Chiao Tung University, 1001 University Road, Hsinchu 30010, Taiwan; 4 Department of Photonics Engineering, Yuan Ze University, 135 Yuan-Tung Road, Taoyuan 32003, Taiwan; 5 Department of Mechanical, Aerospace, and Biomedical Engineering, University of Tennessee, Knoxville, TN 37996, USA; cthsieh@saturn.yzu.edu.tw; 6 Department of Mechanical Engineering, Ming Chi University of Technology, 84 Gongzhuan Road, Taishan District, New Taipei City 243, Taiwan; mjyouh@mail.mcut.edu.tw; 7 State Key Laboratory for Physical Chemistry of Solid Surfaces, Department of Chemistry, Xiamen University, Xiamen 361005, China; qfdong@xmu.edu.cn

**Keywords:** holey structure, graphene nanosheets, nonaqueous electrolyte, composite electrode, volumetric capacitance

## Abstract

We demonstrate a facile and effective method, which is low-cost and easy to scale up, to fabricate holey graphene nanosheets (HGNSs) via ultrafast heating during synthesis. Various heating temperatures are used to modify the material properties of HGNSs. First, we use HGNSs as the electrode active materials for electric double-layer capacitors (EDLCs). A synthesis temperature of 900 °C seems to be optimal, i.e., the conductivity and adhesion of HGNSs reach a compromise. The gravimetric capacitance of this HGNS sample (namely HGNS-900) is 56 F·g^−1^. However, the volumetric capacitance is low, which hinders its practical application. Secondly, we incorporate activated carbon (AC) into HGNS-900 to make a composite EDLC material. The effect of the AC:HGNS-900 ratio on the capacitance, high-rate performance, and cycling stability are systematically investigated. With a proper amount of HGNS-900, both the electrode gravimetric and volumetric capacitances at high rate charging/discharging are clearly higher than those of plain AC electrodes. The AC/HGNS-900 composite is a promising electrode material for nonaqueous EDLC applications.

## 1. Introduction

With the increasing demand for energy storage devices in modern society, supercapacitors (SCs), including electric double-layer capacitors (EDLCs) and pseudocapacitors, represent an attractive energy storage technology owing to their higher power density, wider operating temperature window, superior cycling stability, and greater charge–discharge efficiency compared to secondary batteries [1,2]. EDLCs based on a storage mechanism that employs nonfaradaic charge separation at the electrode–electrolyte interface are currently chosen over pseudocapacitors for practical applications because of their long cycle life, low cost, and high operation voltage [3,4]. Significant efforts have been devoted to the development of new electrode materials and electrolytes for better EDLCs [5,6]. In this field, activated carbon (AC) is currently the most used electrode material due to its high specific surface area, tunable porosity, high packing density, and wide availability [7,8]. However, the low electronic conductivity (due to the low crystallinity and high porosity) and low ionic conductivity (due to the nanosize pores with long diffusion pathways) of AC limit its EDLC performance, especially at high charge–discharge rates [5,9]. The search for new kinds of nanostructured carbon electrode materials such as carbon aerogels, graphene nanosheets (GNSs), carbon nanotubes, ordered mesoporous carbon, and hierarchical porous carbon is a popular research focus [10,11,12].

Among various nanostructured carbons, GNSs are very promising because of their high surface area, great electrical conductivity, excellent mechanical stability, and stable chemical/electrochemical properties [13,14]. A variety of graphene materials such as chemically-modified graphene, porous graphene, microwave-expanded graphene, curved graphene, monolayer or few-layer graphene, etc. have been developed for EDLCs [15,16,17,18,19]. Nevertheless, many previous papers used aqueous electrolytes, which are less practical than nonaqueous electrolytes, which allow higher cell voltage (i.e., ~2.5–3.0 V) [4,20]. It should be noted that the working ions and solvation molecules are different for aqueous and nonaqueous electrolytes [21]. In aqueous electrolytes, the oxygen-containing functional groups on carbon are redox active sites. However, similar redox reactions cannot occur in nonaqueous systems [1]. In addition, the wetting properties of the two kinds of electrolytes are distinct. Some materials that show good wettability with aqueous electrolytes might have poor compatibility with nonaqueous electrolytes [22]. The desirable materials for the two electrolytes can be quite different. Holey graphene nanosheets (HGNSs), with a large number of electroactive sites and efficient pathways for ion transport, are promising for EDLCs. However, the supercapacitive performance of HGNSs in nonaqueous electrolytes has been less well investigated [13,23,24]. There is no systematic performance comparison between AC and HGNSs for nonaqueous EDLC applications. Moreover, many literature papers have focused on the gravimetric capacitances of graphene materials. From a practical point of view, volumetric consideration is also crucial. Thus, both aspects as they relate to AC and HGNS electrodes will be addressed in this work.

HGNSs can not only be the main electrode active materials, but can also act as an electrode additive in EDLCs. Some earlier reports have suggested that combinations of various carbon materials with different structural features and physicochemical properties can give rise to better EDLC performance compared to that of their single-component counterparts due to some synergistic effects [25,26,27]. However, mixing HGNSs with conventional AC particles for improved EDLC properties in nonaqueous electrolytes has seldom been attempted. It is expected that various HGNS incorporation ratios will bring about different capacitive characteristics. This topic is addressed for the first time in this study.

In the present work, we present a facile and effective method, which is low-cost and easy to scale up, to fabricate HGNSs. The effects of the heating temperature on the HGNS material properties are investigated. Applications of HGNSs for the main electrode active material and for mixing with AC particles to modify the electrode performance are both studied. The AC/HGNS composite is found to be a good potential electrode material for nonaqueous EDLCs. With the incorporation of the proper amount of HGNS, both the gravimetric and volumetric capacitances at high charge–discharge rates are significantly higher than those of plain AC electrodes. 

## 2. Experimental Section

### 2.1. HGNS and AC Samples

HGNSs were synthesized using a modified Staudenmaier method [28]. Briefly, natural graphite (Alfa Aesar; particle size: ~70 mm; purity: 99.999%) was chemically oxidized to form graphite oxide (GO). To obtain holey graphene, an ultrafast heating method was used. A tube furnace was set at 300 °C and held for 30 min before GO inlet. To achieve an extremely high heating rate, we rapidly inserted a long-handled stainless-steel spoon containing the GO powder into the center of the quartz tube and immediately flipped the spoon to dump the powder onto the tube surface. By this means, the GO was rapidly reduced, and CO_2_ gas was released at a high rate that built up high pressure between the neighboring graphitic layers. This not only exfoliated the graphite but also punched holes at structurally weak points on the graphene. As a result, holey graphene nanosheets (denoted as HGNS-300) were obtained. These HGNSs were then heated under an argon/hydrogen (5:1) mixed atmosphere at a rate of 60 °C·min^−1^ to 700 °C, 900 °C, 1100 °C, respectively, and held for 1 h. The samples are denoted as HGNS-700, HGNS-900, and HGNS-1100, respectively. The AC powder was supplied by China Steel Chemical Corporation (model ACS20; the specific surface area is ~2000 m^2^ g^−2^) and was used as received. 

### 2.2. Material Characterization

The microstructures were examined using scanning electron microscopy (SEM; FEI Inspect F50, Hillsboro, OR, USA) and transmission electron microscopy (TEM; JEOL 2100F). To study the crystal structures of various samples, X-ray diffractometry (XRD, Bruker, D2 Phaser, Karlsruhe, Germany) was performed. A Raman spectrometer (UniRAM MicroRaman; λ = 532 nm) was used to analyze the carbon bonding characteristics. X-ray photoelectron spectroscopy (XPS, VG Sigma Probe, Waltham, USA) was employed to probe the surface chemical compositions of the samples. The N_2_ adsorption/desorption isotherms were measured at 77 K. The specific surface area was calculated using the Brunauer–Emmett–Teller (BET) method.

### 2.3. Electrochemical Measurements

The electrode slurry was prepared by mixing 80 wt % active material, 10 wt % carbon black, and 10 wt % poly(vinylidenedifluoride) in *N*-methyl-*2*-pyrrolidone solution. The slurry was pasted onto etched Al foil and vacuum-dried at 120 °C for 8 h. The obtained electrode was roll-pressed and punched to match the required dimensions of a CR2032 coin cell. Two symmetrical electrodes divided by a NKK cellulose separator were assembled into a coin cell. The electrolyte was composed of 1 M tetraethylammonium tetrafluoroborate (TEABF_4_; Alfa Aesar, 99 wt %) salt in propylene carbonate (PC; Sigma-Aldrich, 99.7 wt %, St. Louis, MO, USA) solvent. The coin cell assembly was conducted in an argon-filled glovebox (Innovation Technology Co. Ltd.), where both the moisture and oxygen content levels were maintained at below 0.3 ppm. Cyclic voltammetry (CV) and galvanostatic charge–discharge tests were performed using a Solartron 1470E potentiostat within a cell voltage range of 0–2.5 V. For each condition, at least five coin cells were measured. The capacitance deviation was typically less than 5%. The reported data are the median values. Electrochemical impedance spectroscopy (EIS) was used to characterize the cell internal resistance. The frequency range and potential amplitude, regulated with a BioLogic VSP-300 potentiostat, were 10^5^–10^−2^ Hz and 10 mV, respectively. 

## 3. Results and Discussion

**Figure 1:**
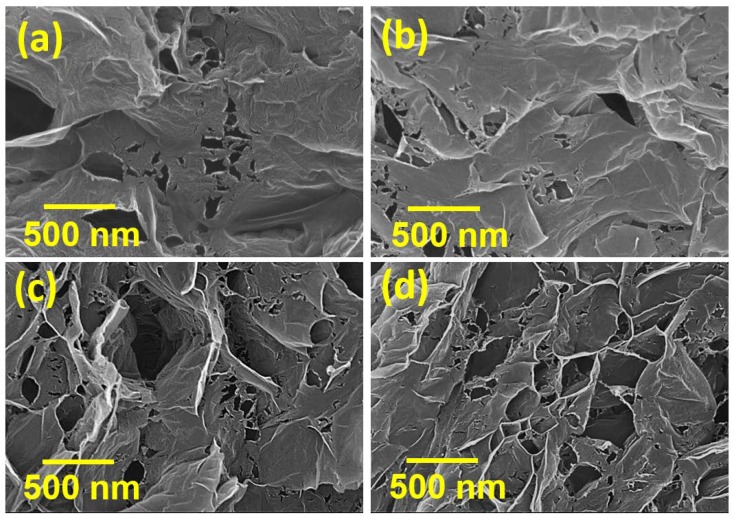
SEM micrographs of (**a**) HGNS-300; (**b**) HGNS-700; (**c**) HGNS-900; and (**d**) HGNS-1100 samples.

Figure 1 shows SEM images of various HGNS samples. Exfoliated carbon nanosheets with submicron-size pores were observed. During the thermal reduction of GO, the exfoliation of neighboring carbon layers resulted from the fast decomposition reaction of the oxygen-containing functional groups. The produced gas built up internal pressure that could overcame the Van der Waals force between the graphitic sheets [29]. This accumulated pressure also pierced holes through the defective regions of the GNSs [30], which were created by strong oxidation during the GO forming step. It was observed that the heating temperature affected the pore structures. A higher temperature enhanced the GO reduction reaction, promoting outgassing, and thus, pore formation. As a result, the holes on the HGNSs increased both in size and in number as the heating temperature was raised.

The TEM images in Figure S1 (Supplementary Materials) reveal the high electron transparency of the HGNS samples. This indicates that the graphene nanosheets are composed of only a few carbon layers. The wrinkles of the HGNSs are ascribed to the existence of sp3-hybridized carbon, which causes local structural nonuniformity [31]. The results confirm that this high-heating-rate process is an economical and effective way to synthesize holey-type GNSs. This method is template-free, residual-free, and easily scalable.

**Figure 2:**
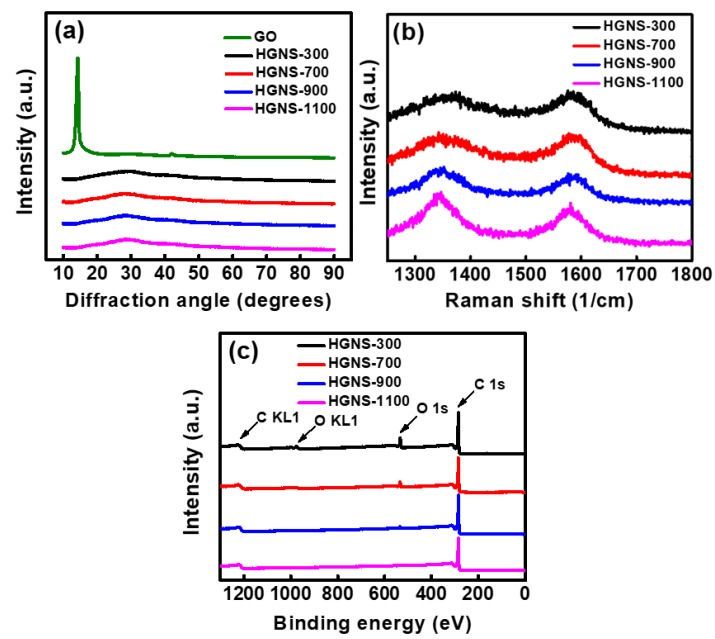
(**a**) XRD pattern; (**b**) Raman spectra; and (**c**) XPS spectra of various samples.

The crystallinity of various samples was characterized by XRD. In Figure 2a, the GO sample shows a characteristic peak at a 2θ angle of 11.8°, indicating a significantly enlarged interlayer distance due to the existence of oxygen-containing groups between the graphitic layers. However, after thermal reduction, this diffraction peak disappears, and only an extremely broad and weak peak is found at around 27°. These results indicate that the oxygen-containing functional groups were reduced. At the same time, the graphite layers peeled off [32], losing the ordering structure along the c axis. It was found that all the HGNS samples exhibited similar XRD patterns. A temperature of 300 °C is sufficient to exfoliate the GO, producing few-layer graphene nanosheets.

Raman spectroscopy was employed to investigate the carbon bonding structures of various HGNS samples. The in-plane vibration of the graphitic lattice correlates to the G band, whereas the disorders such as impurity, imperfection, and sp2 domain boundaries are associated with the D band [33]. The D and G bands were found at ~1360 and ~1585 cm^−1^, respectively. 

As shown in Figure 2b, the D-band intensity gradually increases with increasing the heating temperature. The D-to-G band intensity ratio (ID/IG) calculated from the Raman spectra is indicative of the degree of structural defect in the HGNSs. As shown in Table 1, the ID/IG ratio increases monotonously from 1.01 for HGNS-300 to 1.18 for HGNS-1100. These results can be attributed to the increase in defect sites along the hole edges created at higher temperature. Table 1 also shows the chemical compositions of the HGNS samples evaluated using an elemental analyzer. The oxygen content decreases from 16.3 to 2.2 at % when the temperature increases from 300 to 1100 °C. No other impurity element was detected in these analyses.

XPS was also used to examine the surface chemistry of the HGNSs. The survey scans in Figure 2c show that besides carbon and oxygen, no other element can be detected for all the samples. It can be seen that as the reduction temperature increases, the intensity of the oxygen peak decreases. This confirms that higher temperatures can more effectively remove the oxygen-containing functional groups on HGNS surface. The data are consistent with the results (in Table 1) obtained from the elemental analyzer. A representative high-resolution XPS C 1s spectrum of HGNS-900 is shown in Figure S2 (Supplementary Materials), which indicates that oxygen exists in the C–OH, C=O, and COOH forms. The synthesis temperature affected both the porosity and surface functionality of the HGNSs.

**Figure 3:**
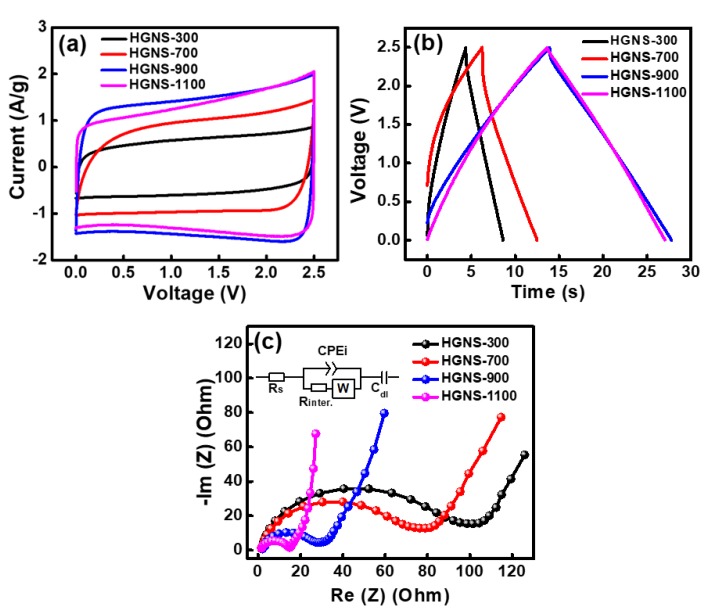
(**a**) CV curves of various HGNS cells measured at a potential sweep rate of 50 mV·s^−1^; (**b**) Galvanostatic charge–discharge curves of various HGNS cells measured at 5 A·g^−1^; (**c**) EIS Nyquists plots of various HGNS cells.

In order to evaluate the electrochemical properties of various HGNS electrodes, symmetrical cells with a TEABF4/PC electrolyte were assembled. Figure 3a shows the obtained CV curves measured at a potential sweep rate of 50 mV·s^−1^. It can be observed that the HGNS-900 and HGNS-1100 electrodes exhibited more rectangular CV curves, indicating better capacitive behavior compared to the HGNS-300 and HGNS-700 electrodes. It is believed that the removal of the oxygen-containing functional groups on HGNSs can reduce the electrode internal resistance, promoting electrochemical performance. Figure 3a also shows that the CV enclosed area, which corresponds to the energy storage amount, increases monotonously with the heating temperature up to 900 °C, and slightly decrease at 1100 °C. Clearly, the heating temperature is a matter of HGNS capacitance. Figure 3b shows the charge–discharge curves of various cells measured at 5 A·g^−1^. The HGNS-300, HGNS-700, HGNS-900, and HGNS-1100 electrodes exhibited specific capacitances of 17, 25, 56, and 53 F·g^−1^, respectively. A temperature of 900 °C appeared to achieve capacitance optimization. It was reported that the oxygen-containing functional groups can contribute some pseudocapacitance [34,35]. We speculate that since few of these groups were left on the HGNS-1100 surface, the specific capacitance slightly decreased.

Figure 3c shows the EIS spectra of various cells. The Nyquist plots are characterized by a semicircle at high frequency and a straight line at low frequency [36]. The diameter of the semicircle can be mainly attributed to the interface contact resistance (R_inter._) between the HGNSs [37]. Table S1 (Supplementary Materials) shows the R_inter._ values of various cells. The HGNS-300 and HGNS-700 cells exhibited R_inter._ values of 100 and 75 Ω, respectively. The high resistance is responsible for the unsatisfactory electrochemical properties shown in Figure 3a,b. Increasing the temperature beyond 700 °C effectively reduced the R_inter._, which is in line with the oxygen concentration variation trend shown in Table 1. The results indicate that the reduction of oxygen-containing functional groups on HGNSs can decrease the contact resistance between graphene sheets. The R_inter._ values for the HGNS-900 and HGNS-1100 cells were 26 and 16 Ω, respectively.

It was observed that the active material coating layer of the HGNS-1100 electrode was not as uniform as the others, and its adhesion with the Al current collector was relatively poor. While casting the electrode slurry with a doctor blade gap of 50 µm, the final thickness of the HGNS-1100 layer after drying was ~9 µm, which is considerably lower than that (~30 µm) of the other electrodes. This is probably related to the fact that the oxygen-containing functional groups had been removed to a greater degree. Thus, the bonding force between the HGNSs became weaker. Moreover, the HGNS-1100 film density was lower (~0.140 g·cm^−3^) than those of the other electrodes, as shown in Table S2 (Supplementary Materials). The relatively poor adhesion, low thickness, and low film density make the HGNS-1100 unfavorable for practical applications. Based on above results, HGNS-900 is more promising, especially when energy consumption during material synthesis is a relevant factor.

In the literature, high gravimetric capacitances (e.g., >120 F·g^−1^) of GNSs were reported for thin electrodes [38,39]. However, such thin electrodes are unlikely to be useful, and a practical coating thickness should be at least dozens of microns. Our data indicate that even with the holey structure, the HGNS-900 exhibited a gravimetric capacitance ~56 F·g^−1^ at 30 µm thickness. One reason for the low capacitance is the restacking nature of graphene sheets for a thick electrode [40,41]. Another drawback of HGNS-900 is the much lower film density compared to that of AC (0.156 vs. 0.333 g·cm^−3^). Accordingly, the HGNS-900 alone is not an ideal electrode material for EDLCs.

**Figure 4:**
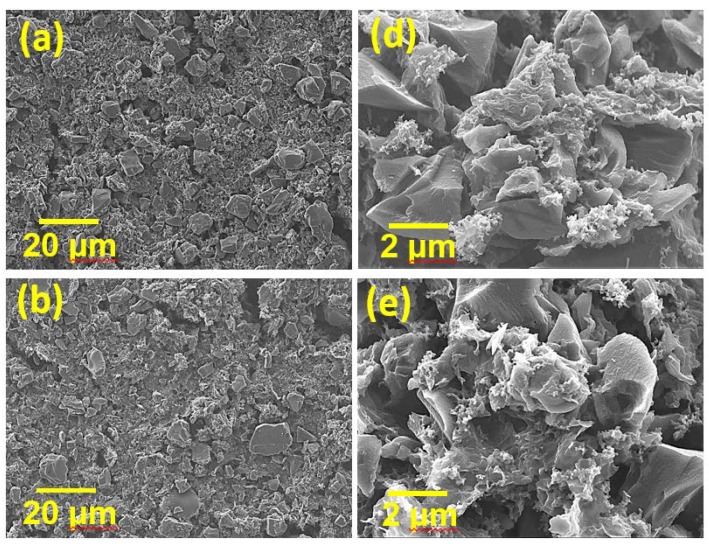

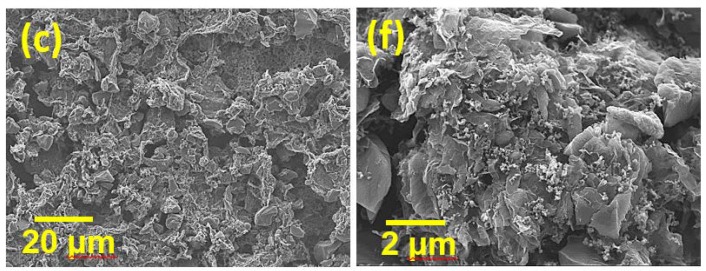
Low-magnification SEM images of (**a**) AC/HGNS-900 (20:1); (**b**) AC/HGNS-900 (10:1); and (**c**) AC/HGNS-900 (5:1) electrodes. High-magnification SEM images of (**d**) AC/HGNS-900 (20:1); (**e**) AC/HGNS-900 (10:1); and (**f**) AC/HGNS-900 (5:1) electrodes.

However, mixing HGNS-900 with AC is worthy of consideration. AC has high specific surface area (2000 vs. 550 m^2^·g^−1^ for HGNS-900), high density, and low cost; however, its electronic conductivity is not sufficient. We speculated that the incorporation of HGNS-900 may modify the properties of the AC electrode. In this section, AC was mixed with HGNS 900 in various proportions (AC:HGNS-900 weight ratios of 20:1, 10:1, 5:1). The surface morphologies of the electrodes were examined by SEM; the obtained low-magnification images are shown in Figure 4a–c. For the AC/HGNS-900 (20:1) electrode, the proportion of AC was high, so the electrode was mostly composed of AC particles. Since AC particles are granular, the surface was slightly uneven and discontinuous. For the AC/HGNS-900 (10:1) electrode, the active material layer appeared to be relatively compact and continuous because HGNS-900 was uniformly dispersed between the AC particles. However, when the HGNS-900 amount further increased, the AC/HGNS-900 (5:1) electrode morphology became loose and nonuniform (with agglomeration). Moreover, the active material particles were not closely connected, with many voids in between. Figure 4d–f show high-magnification SEM images of the electrodes. It was observed that at a low HGNS-900 ratio (i.e., the 20:1 and 10:1 electrodes), the HGNSs were dispersed between the AC particles, acting as conducting bridges. When the HGNS-900 ratio increased (i.e., the 5:1 electrode), the AC particles were densely covered, and agglomeration of the graphene sheets occurred.

**Figure 5:**
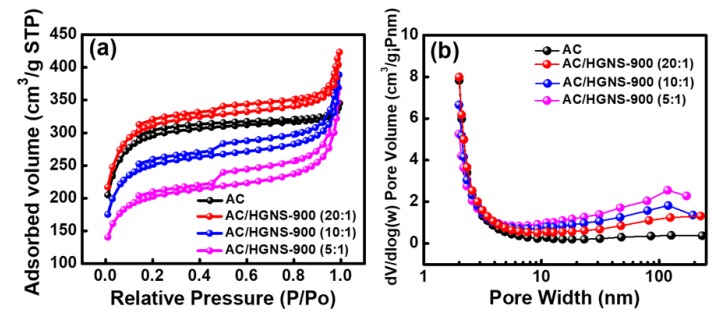
(**a**) N2 adsorption/desorption isotherms and (**b**) pore size distribution data of various electrode coating layers.

The surface area plays a vital role in determining the EDLC performance. In order to better understand the effects of HGNS-900 incorporation on the electrode specific surface area, N_2_ adsorption/desorption measurements of the electrode coating layers were performed. As shown in Figure 5a, the calculated BET surface area of the AC electrode was 917 m^2^·g^−1^, which is considerably lower than that of AC powder (i.e., 2000 m^2^·g^−1^). This reflects the fact that the AC particles agglomerated after slurry coating and drying. Additionally, the electrode binder may block the pores of AC, thereby reducing the surface area. The surface area for AC/HGNS-900 (20:1) reached 968 m^2^ ·g^−1^, indicating that the incorporation of HGNS could alleviate the AC aggregation and pore blocking problems. However, with further increasing the HGNS-900 ratio, the BET surface area decreased (e.g., 784 m^2^·g^−1^ for AC/HGNS-900 (10:1)) because the intrinsic specific surface area of HGNS-900 is lower than that of AC. The surface area of AC/HGNS-900 (5:1) was reduced to 635 m2·g^−1^, since the AC surface was covered by agglomerated graphene sheets that shielded the AC pores (see Figure 4f). Figure 5b shows the pore size distribution of various electrodes. It was confirmed that the incorporation of HGNS can create meso- and macro-pores between AC particles. Nevertheless, if the proportion of the macro-pores is too high (i.e., AC/HGNS-900 (5:1)), the electrode becomes loose and discontinuous (see Figure 4c).

**Figure 6:**
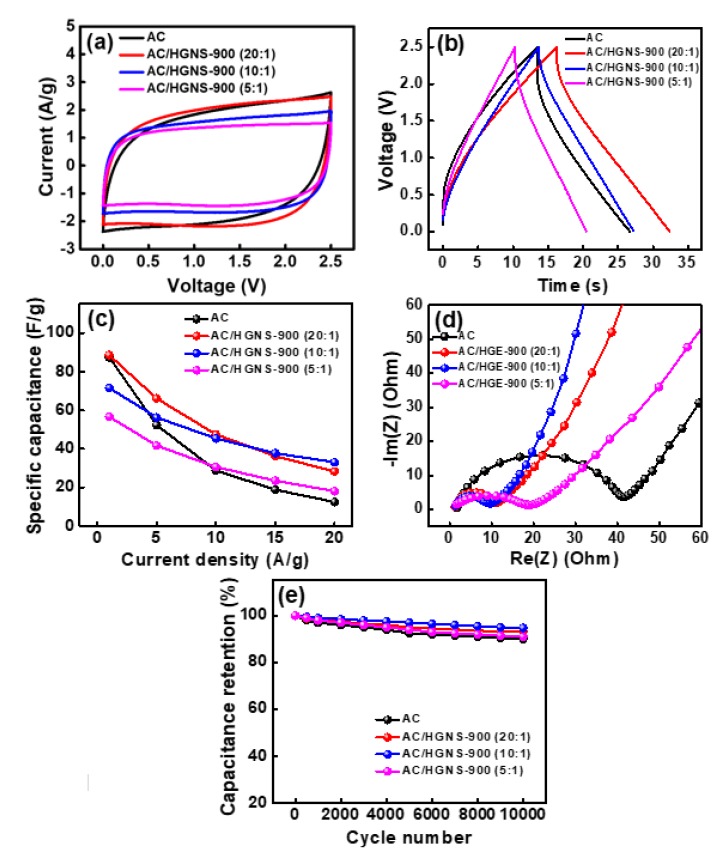
(**a**) CV curves of various cells measured at potential sweep rate of 50 mV·s^−1^; (**b**) Galvanostatic charge–discharge curves of various cells measured at 5 A·g^−1^; (**c**) Specific capacitances of various electrodes at different current densities; (**d**) EIS Nyquists plots and (**e**) cycling stability data of various cells.

The electrochemical properties of various AC/HGNS-900 cells were evaluated by CV at a potential scan rate of 50 mV·s^−1^; the obtained data are shown in Figure 6a. Clearly, the incorporation of HGNS-900 can decrease the electrode internal resistance and make the CV shape more rectangular. Figure 6b shows the galvanostatic charge–discharge curves of the cells measured at 5 A·g^−1^ (the curves measured at different current densities are shown in Figure S3). The calculated specific capacitances for AC, AC/HGNS-900 (20:1), AC/HGNS-900 (10:1), and AC/HGNS-900 (5:1) electrodes are 52, 66, 56, and 42 F·g^−1^, respectively, at 5 A·g^−1^. The specific capacitances of various electrodes evaluated at different current densities (from 1 to 20 A·g^−1^) are shown in Figure 6c and Table S3 (Supplementary Materials). An electrode property comparison with the literature data is shown in Table S4 (Supplementary Materials). At relatively low rates (up to 10 A·g^−1^), the optimal AC:HGNS-900 ratio seems to be 20:1, because the electrode surface area and conductivity have been improved (vs. the plain AC electrode) at the same time. The AC:HGNS-900 ratio of 10:1 is more favorable for higher rate applications (>10 A·g^−1^), revealing that the incorporation of HGNS can effectively promote the electrode charge–discharge kinetics. A proper amount of meso- and macro-pores (see Figure 5b) is beneficial for electrolyte penetration, also enhancing the ion transport. However, when the HGNS-900 content is excessive (i.e., AC:HGNS-900 ratio of 5:1), adverse effects on the capacitance and electrode rate capabilities were observed. The Ragone plots for various cells are shown in Figure S4 (Supplementary Materials) to further express these ideas.

Figure 6d and Table S5 (Supplementary Materials) compare the EIS data of various cells. The R_inter._ of the AC cell is 40 Ω. With the incorporation of HGNS, the AC/HGNS-900 (20:1) and AC/HGNS-900 (10:1) cells showed R_inter_ values of 15 and 10 Ω, respectively. The HGNSs act as the conducting pathways that surround and connect the AC particles, reducing the electrode resistance. This explains the improved high-rate performance of these electrodes. However, when the AC:HGNS-900 ratio became 5:1, the R_inter._ increased to 20 Ω. This can be attributed to the discontinuity of the electrode layer, as shown in Figure 4c. The excessive HGNSs agglomerated, and thus separated the AC particles, resulting in increased interparticle resistance.

The durability of supercapacitors is a crucial factor for practical applications. Figure 6e compares the cycling stability of AC and various AC/HGNS-900 cells. The measurements were carried out with a charge–discharge rate of 5 A·g^−1^. As shown, the capacitance retention ratios for all electrodes were 91–95% after 10,000 cycles. The Incorporation of HGNS did not significantly affect the electrode cycling stability. 

Table 2 shows the film density values, gravimetric capacitances, and volumetric capacitances of various electrodes. Mixing HGNS-900 with AC to optimize the electrode performance seems to be more promising than using HGNS-900 alone as the electrode active material. As expected, the film density decreased with increasing the HGNS-900 ratio. The AC/HGNS-900 (5:1) is less appealing, especially when volumetric performance is concerned. Accordingly, the incorporation of about 5–10 wt % HGNS-900 is a reasonable design to improve the high-rate EDLC performance of the AC electrode. This ratio can be tuned to meet various gravimetric and volumetric capacitance requirements under various operation current densities.

## 4. Conclusions

A cost-effective method exploiting ultrafast heating during GO reduction was employed to produce HGNSs. It was found that the heating temperature played a role in determining the internal resistance, capacitance, and processability of the HGNS electrodes. However, even for the optimal HGNS-900 electrode, the specific capacitance is still unsatisfactory (56 F·g^−1^) and the film density is inadequate (0.156 g·cm^–3^) at a practical electrode thickness of 30 µm. It was concluded that HGNS-900 alone is not a competitive electrode active material. However, HGNS-900 can be an effective performance promoter for EDLC AC electrodes. The AC:HGNS-900 ratio is a crucial factor affecting the distribution of HGNSs within the electrode, and thus, the resulting charge–discharge performance. If the HGNS-900 is excessive, the HGNSs agglomerated, shielded the AC pores, and separated the AC particles, deteriorating the electrode performance. However, a proper amount of HGNS-900 can alleviate the AC aggregation and create conducting pathways within the electrode. As a result, both the gravimetric and volumetric capacitances at high charge–discharge rates are considerably higher than those of the plain AC electrode. The appealing performance of the AC/HGNS-900 composite has shown great potential for high-performance energy storage applications.

## Figures and Tables

**Table 1:** Raman *I*_D_/*I*_G_ ratio and chemical compositions of various HGNS samples evaluated using an elemental analyzer.

Sample	*I*_D_/*I*_G_	*C* (at %)	*O* (at %)
HGNS-300	1.01	83.7	16.3
HGNS-700	1.02	88.5	11.5
HGNS-900	1.07	96.0	4.0
HGNS-1100	1.18	97.8	2.2

**Table 2:** Film densities, gravimetric capacitances, and volumetric capacitances of various AC/HGNS electrodes.

Sample	Thickness (μm)	Film Density (g·cm^−3^)	Current Density			
			1 A·g^−1^	20 A·g^−1^	1 A·g^−1^	20 A·g^−1^
			Gravimetric Capacitance (F·g^−1^)		Volumetric Capacitance (F·cm^−3^)	
AC	32	0.333	87	12	29.0	4.0
AC/HGNS-900 (20:1)	32	0.322	89	28	28.7	9.0
AC/HGNS-900 (10:1)	32	0.312	72	33	22.5	10.3
AC/HGNS-900 (5:1)	32	0.260	57	18	14.8	4.7

## Supplementary Materials

The following are available online at https://www.mdpi.com/2073-4360/12/4/765/s1. Table S1- Electrolyte resistance (Rs) and interface contact resistance (Rinter.) values of various HGNS cells. Table S2- Thickness and film density values of various HGNS electrodes. Table S3- Gravimmetric capacitance of AC, HGNS-900 and various AC/HGNS-900 electrodes. Table S4- Performance comparison of previously reported AC/graphene composite electrodes and AC/HGNS-900 (20:1). Table S5- Electrolyte resistance (Rs) and interface contact resistance (Rinter.) values of various cells. Figure S1- TEM micrographs of HGNS samples. Figure S2- XPS C1 s spectra of HGNS-900 sample, Figure S3- Galvanostatic charge–discharge curves of AC and various AC/HGNS-900 cells measured at various current densities. Figure S4- Ragone Plots of various cells.
