# Supplementary material for: A Holey Graphene Additive for Boosting Performance of Electric Double-Layer Supercapacitors
Source: Polymers (Basel). 2020 Apr 1;12(4):765. doi: 10.3390/polym12040765 (PMC7240531; doi:10.3390/polym12040765)
Supplement: Supplementary file 1 [file polymers-12-00765-s001.pdf]

Supplementary Information

A holey graphene additive for boosting performance of electric double-layer supercapacitors

Jun-Bin Huang, Jagabandhu Patra, Ming-Hsien Lin, Ming-Der Ger, Yih-Ming Liu, Nen-Wen Pu, Chien-Te Hsieh, Meng-Jey Youh, Quan-Feng Dong, Jeng-Kuei Chang

Table S1. Electrolyte resistance (R_s) and interface contact resistance ($R_{inter.}$) values of various HGNS cells derived from Figure 3 (c).

Sample	R_s (Ω)	$R_{inter.}$ (Ω)
HGNS-300	1.5	100
HGNS-700	1.5	75
HGNS-900	1.7	26
HGNS-1100	1.6	16

Table S2. Thickness and film density values of various HGNS electrodes.

Sample	Thickness (μm)	Film density (g cm^{-3})
HGNS-300	30	0.157
HGNS-700	30	0.157
HGNS-900	30	0.156
HGNS-1100	9	0.140

Table S3. Gravimetric capacitances of AC, HGNS-900, and various AC/HGNS-900 electrodes.

	Capacitance (F/g)				
Current rate (A/g)	AC	HGNS-900	AC/HGNS-900 (20:1)	AC/HGNS-900 (10:1)	AC/HGNS-900 (5:1)
0.5	95	63	92	75	60
1	87	61	89	72	57
5	52	56	66	56	42
10	28	48	48	45	31
15	19	43	36	38	24
20	12	38	28	33	18

Table S4. Performance comparison of previously reported AC/graphene composite electrodes and AC/HGNS-900 (20:1) in this study.

Electrode material	Electrolyte	Electrode thickness	Film density	Capacitance	Retention after cycling	Reference
AC/Graphene	1-ethyl-3-methylimidazolium tetrafluoroborate	NA	NA	94 F/g at 0.1 A/g	89% after 3000 cycles	1
AC/Porous graphene	1 M $TEABF_4$/PC	NA	~0.3 g/cm^3	103 F/g at 200 mV/s	94.7% after 5000 cycles	2
KOH activated AC/Graphene	1 M $TEABF_4$/Acetonitrile	NA	NA	173 F/g at 2 A/g	NA	3
AC/Graphene	1M $LiPF_6$ solution	NA	NA	19.45 F/g at 1 mV/s	NA	4
AC/Graphene hybrid aerogel	1 M $TEABF_4$/PC	NA	NA	144 F/g at 0.05 A/g	NA	5
AC/Graphene oxide	1 M $TEABF_4$/PC	NA	NA	26.87 F/g at 0.1 A/g	~100% after 500 cycles	6
AC/CNT/RGO	1M $LiClO_4$ in Ethylene carbonate/Diethyl carbonate	NA	NA	101 F/g at 0.2 A/g	75% after 1000 cycles	7
**AC/HGNS-900 (20:1)**	**1 M $TEABF_4$/PC**	**32 μm**	**0.32 g/cm^3**	**92 F/g at 0.5 A/g**	**93% after 10000 cycles**	**This work**

Table S5. Electrolyte resistance (R_s) and interface contact resistance ($R_{inter.}$) values of various cells derived from Figure 6 (d).

Sample	R_s (Ω)	$R_{inter.}$ (Ω)
AC	1.7	40
AC/HGNS-900 (20:1)	1.6	15
AC/HGNS-900 (10:1)	1.5	10
AC/HGNS-900 (5:1)	1.5	20

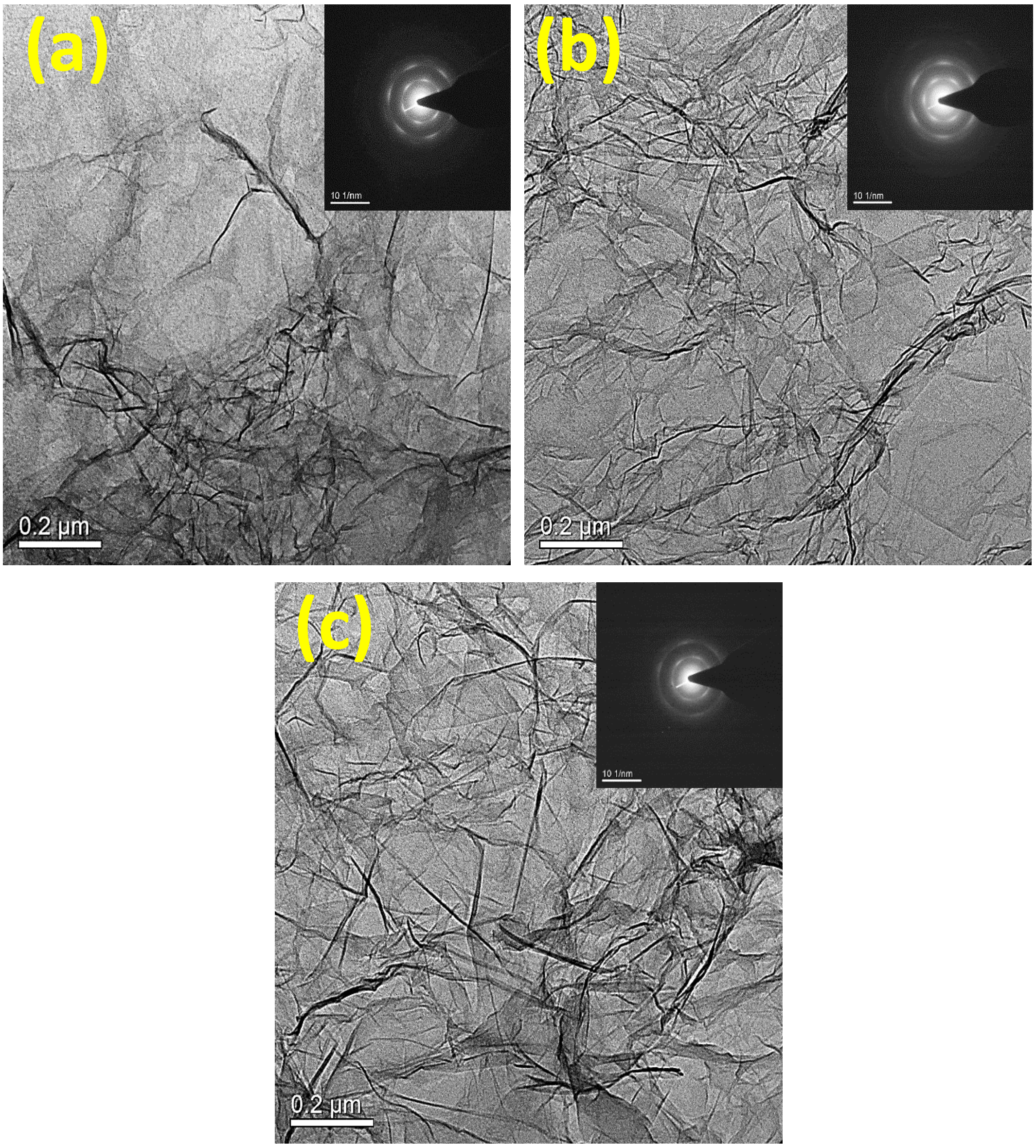

Figure S1. TEM micrographs of (a) HGNS-700, (b) HGNS-900, and (c) HGNS-1100 samples.

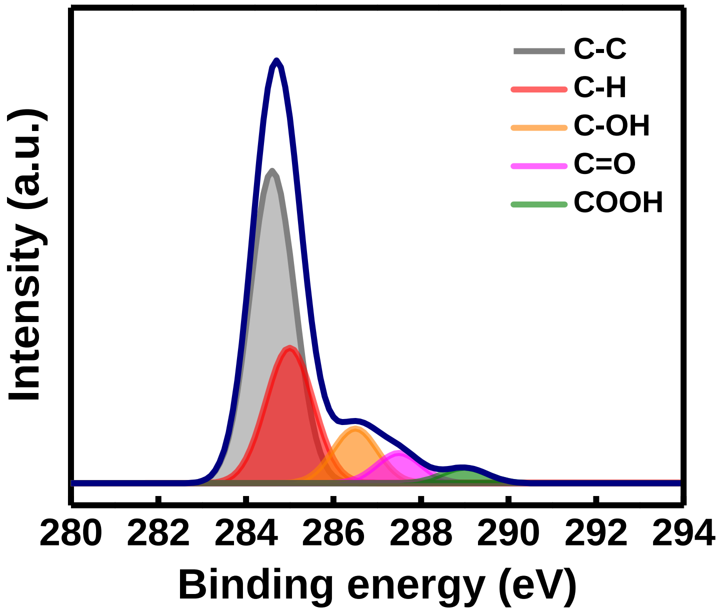

Figure S2. XPS C1 s spectrum of HGNS-900 sample.

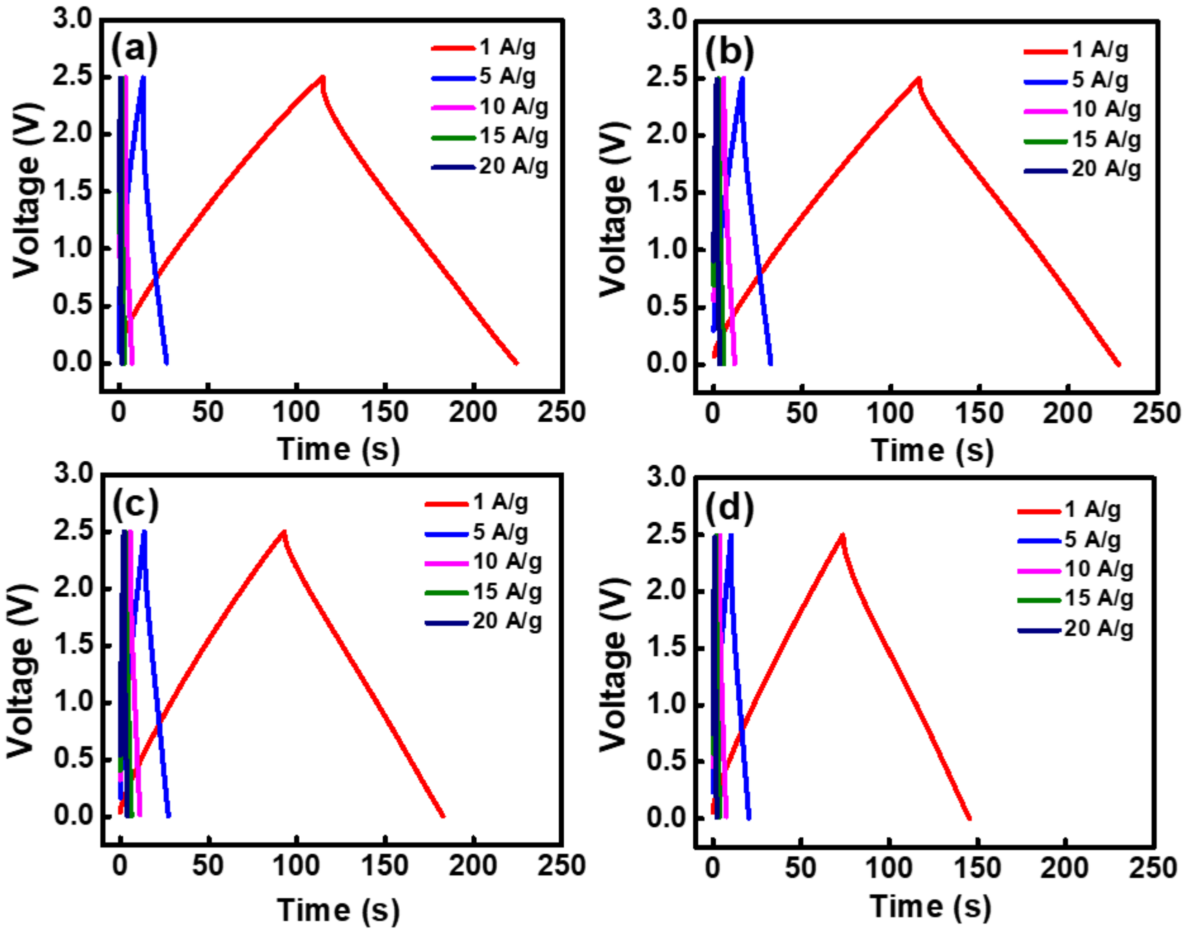

Figure S3. Galvanostatic charge-discharge curves of (a) AC, (b) AC/HGNS-900 (20:1), (c) AC/HGNS-900 (10:1), and (d) AC/HGNS-900 (5:1) cells measured at various current densities.

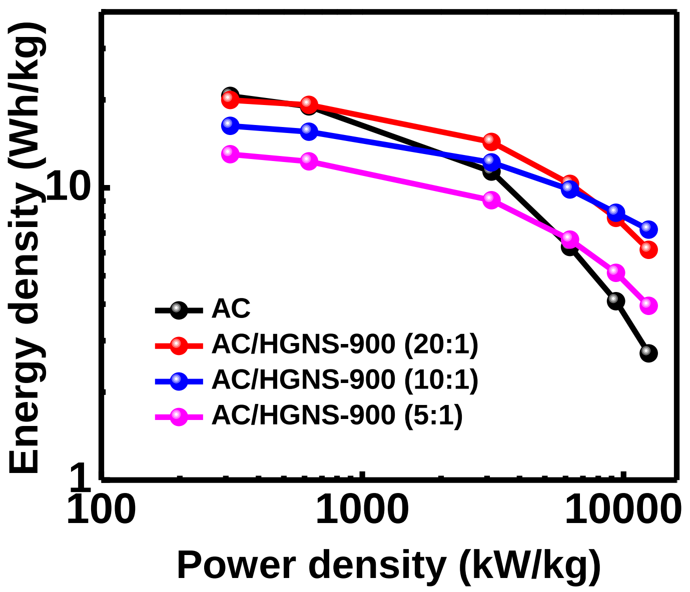

Figure S4. Ragone plots of various cells calculated based on various charge-discharge current densities.

Reference

1. Chen, Y.; Zhang, X.; Zhang, H.; Sun, X.; Zhang, D.; Ma, Y. High-performance supercapacitor based on a graphene-activated carbon composite prepared by chemical activation, *RSC Adv*. **2012**, *2*, 7747–7753.
2. Zheng, C.; Zhou, X.; Cao, H.; Wang, G.; Liu, Z. Synthesis of porous/activated carbon composite with high packing density and large specific area for supercapacitor electrode material, *J Power Sources* **2014**, *258*, 290–296.
3. Yu, S.; Li, Y.; Pan, N. KOH activated carbon/graphene nanosheets composites as high performance electrode materials in supercapacitors, *RSC Adv*. **2014**, *4*, 48758–48764.
4. Azam, M. A.; Dorah, N.; Seman, R. N. A. R.; Manaf, N. S. A.; Kudin, T. I. T. Electrochemical performance of activated carbon and graphene based supercapacitor, *Mater. Technol.* **2015**, *30*, A14–A17.

5. Zhu, Q.; Ma, L.; Wang, H.; Jia, M.; Guan, Y.; Xu, B. Activated carbon/graphene hybrid aerogel as electrode materials for high performance supercapacitors, *ChemistrySelect* **2017**, *2*, 4456–4461.
6. Du, W.; Lv, Y; Lu, H.; Chen, Z.; Wright, D. S.; Zhang, C. Surface modification by graphene oxide: an efficient strategy to improve the performance of activated carbon based supercapacitors, Chinese Chem. Lett. **2017**, *28*, 2285–2289.
7. Li, X.; Tang, Y.; Song, J.; Yang, W.; Wang, M.; Zhu, C.; Zhao, W.; Zheng, J.; Lin, Y. Self-supporting activated carbon/carbon nanotube/reduced graphene oxide flexible electrode for high performance supercapacitor, Carbon, **2018**, *129*, 236–244.
